# Ozone Therapy in the Management and Prevention of Caries

**DOI:** 10.7759/cureus.37510

**Published:** 2023-04-12

**Authors:** AbdulMajeed A AlMogbel, Maram I Albarrak, Shahad F AlNumair

**Affiliations:** 1 Orthodontics and Pediatric Dentistry Department, College of Dentistry, Qassim University, Buraydah, SAU; 2 Family Dentistry Department, National Guard Hospital, Riyadh, SAU; 3 Dental Surgery Department, College of Dentistry, Qassim University, Buraydah, SAU

**Keywords:** ozonated water, ozone-oxygen gas, review article, dentistry, ozone therapy

## Abstract

The article covered the function and results of ozone treatment in managing and preventing dental caries. Specifically, the author examined ozone and its benefits, including bactericidal, analgesic, anti-inflammatory, and immunomodulatory effects. Ozonated water, ozonated olive oil, and ozone gas are forms of ozone used in dentistry. The authors provided examples of studies regarding the positive impact of ozone therapy on patients with caries. Also, the research authors described several effects of ozonated water, namely, disinfectant, anti-inflammatory, activation of intracellular metabolism of the oral mucosa and dental wounds, advancement of local blood circulation, provocation of regenerative functions, and hemostatic effect in capillary bleeding. The ozone generator and equipment for creating an ozone-oxygen (O3/O2) gas mixture were mentioned as required in dentistry to produce ozone.

## Introduction and background

Non-drug methods of caries lesion treatment attract numerous supporters due to the high level of allergization of the population, the manufacturing of artificial materials to cure dental caries, and the high cost of an esthetic filling. The use of ozone-oxygen mixtures is a qualitatively new solution to urgent problems in treating many diseases. Ozone therapy, which has been used to treat and prevent diseases of internal organs for many years, has found application in various areas of medicine, including gynecology, urology, immunology, venereology, cosmetology, and dentistry. This treatment method can be combined with other techniques or used separately. Ozone therapy is based on the use of an ozone-oxygen mixture as a therapeutic effect, which is a component present in the environment. Ozone therapy is characterized by ease of use, high efficiency, and good tolerability [1].

## Review

Ozone and its benefits

Ozone, as an allotropic form of oxygen, is best known for maintaining the ecological balance on Earth and protecting living organisms from exposure to ultraviolet rays. Ozone is a gas with a specific smell. Currently, the following properties of ozone are best studied: its bactericidal, virucidal, and fungicidal action; its activation of metabolism; its anti-hypoxic effect; its optimization of pro- and antioxidant systems; its detoxification effect; its anti-inflammatory effect; its dose-dependent effect on the proteolytic systems of the body; its pain relief effect; and its immunomodulatory properties [1].

Among the biological effects of ozone, its bacterio-, fungi-, and virucidal effects are traditionally considered the most crucial. These direct effects of ozone manifest in the external application of its various modifications, especially in high concentrations. Moreover, unlike many well-known antiseptics, ozone does not irritate nor destroy human integumentary tissues because, in contrast to microorganisms, a multicellular human organism has a powerful antioxidant defense system [1].

Following microbiological research by Oliver et al. (2019) [2], ozone can diminish all identified categories of gram-positive and gram-negative bacteria, particularly *Pseudomonas aeruginosa* and *Legionella*; all lipo- and hydrophilic viruses, among which hepatitis A, B, and C can be mentioned; spores; and vegetative types of existing and discovered pathogenic fungi and protozoa [2]. The bactericidal effect of ozone is explained by the violation of the integrity of bacterial cell membranes caused by the oxidation of phospholipids and lipoproteins. Almaz and Sönmez [3] also found that ozone penetrates into the microbial cell, reacts with the substances of the cytoplasm, and transforms the closed DNA plasmid into open DNA, which reduces the proliferation of bacteria.

Thus, ozone has a bactericidal, analgesic, anti-inflammatory, and immunomodulatory effect. Today, special equipment is produced for water ozonation (ozonizers), which creates an ozone-oxygen (O3/O2) gas mixture. Water is used to flush the mouth, rinse and irrigate wounds, and treat the carious area with a gas mixture. Internal use is also possible. For example, it can be used in case of intoxication, and intravenous drip infusions are prescribed.

The use of ozone in dentistry

Dental caries is a multifactorial infectious disease characterized by the demineralization of hard tissues, which, under certain conditions, can lead to the development of carious cavities [4]. Bacteria play an essential role in initiating and developing caries. Reducing the level of cariogenic bacteria in plaque and the carious cavity is one measure for preventing and treating dental caries. To prevent the development of secondary caries, which may be associated with residual bacteria under the restorations, the high-quality disinfection of the carious cavity is critical.

Mohammadi et al. [5] have defined three crucial forms of application of ozone to oral tissues: ozonated water, ozonated olive oil, and ozone gas. These forms may be used alone or in combination to treat dental conditions. Studies have shown the widespread use of ozone in various areas of dentistry, including maxillofacial surgery, endodontics, and pediatric therapy [1,3,5].

The ozone-oxygen (O3/O2) mixture is delivered hermetically through a cord and a cap into the carious cavity of the tooth under pressure, with the specific concentration and exposure time necessary to destroy microorganisms in the affected area of the tooth prescribed by the doctor [6]. Rickard et al. [7] explain that the ozone generator provides both the supply and suction of the used oxygen: through one of the connecting tubes in the flexible cord, the concentration prescribed by the doctor is supplied under pressure, and through the other tube, the used O3/O2 is simultaneously pumped into the thermoelectric destructor using an ozone-resistant vacuum pump. The procedure occurs several times for 1-2 minutes at room temperature. Caps in one of five sizes are placed tightly on a specific tooth before the procedure to seal the tooth’s treated cavity and ensure that ozone does not leak and enter the respiratory tract of the patient or doctor. Using caps of various sizes made of elastic, resilient material and the vacuum under the cap and around the tooth, created by a special ozone-resistant vacuum pump, ensures that ozone does not enter the human respiratory tract.

When used for just a few minutes, ozone is guaranteed to destroy bacteria that cause caries. By penetrating into the fissures and dentinal tubules, ozone has a calming effect on the nerve endings of the sensory organs and stimulates blood flow. Therefore, ozone is an excellent pain reliever. A et al. [8] have highlighted how, depending on the type of bacteria and degree of tooth damage, the doctor can sensibly prescribe the concentration of ozone in the O3/O2 and the time of tooth treatment. At the same time, 99.9% of the bacteria that cause caries are destroyed when exposed to ozone [8]. Over the weeks following treatment, the treated tooth remineralizes, after which it becomes hard and resistant again. The use of ozone in combination with remineralizing agents facilitates the complete removal of the carious lesion [9-11].

In an eight-month study by Dähnhardt et al. [12] on the effectiveness of ozone for treating dental caries in children with dental phobia, a decrease in the feeling of fear was found in children treated with ozone. The use of ozone for the noninvasive treatment of dentin caries in preschool-age children with dental phobia significantly reduces the discomfort of children during treatment without reducing the quality of filling carious cavities during a six-month period. Studies by Baysan and Lynch [13] and Johansson et al. [14] have demonstrated the antimicrobial effect of ozone: a significant reduction in *Streptococcus mutans* and *Streptococcus sobrinus* in the oral cavity and carious cavity after exposure to ozone for 10-20 seconds in the treatment of root caries and cervical caries in children, and no effect on *Lacticaseibacillus casei* [15-18]. In separate studies, Huth et al. [19] and Zaura et al. [20] have described the results of the complete disappearance of carious spots under the influence of ozone. However, the registration of the results in the structure of such studies was primarily conducted according to subjective assessment criteria without implementing approaches using specific indices or adapted numerical indicators.

In dentistry, two types of ozone therapy devices are commonly used. The first one is the apparatus for the ozonation of water. Highly ozonated water is used for sterilization by the flow method. The ozone concentration in such water ranges from 10 to 100 μg/mL because different treatment methods require significant differences in ozone concentration [21]. Electric ozonizers of this type are not only popular but also reliable, easy, and safe to use. The second type is the apparatus for creating an ozone-oxygen gas mixture. This equipment is also popular and in demand. The exposure time during treatment usually does not exceed 30 seconds [21]. An ozone-oxygen mixture under pressure is fed into the carious cavity or the periodontal pocket, which allows for achieving the maximum therapeutic effect.

A modern dental ozone generator allows for the destruction of all microorganisms in the treated area with 99.9% reliability [8]. With regard to the initial stages of caries development, ozone therapy can stop the process without the need to fill minor defects. In the area treated with ozone, only the tooth enamel is coated with a special composition that strengthens its walls and completely stops the further development of caries. Ozone therapy is a proper addition to traditional treatment methods in dentistry, which allows for the reduction of the number of drugs and, in some cases, completely prevents the need for their use (for example, painful injections of an antibiotic into the gums).

The effects of ozone and ozone therapy in dentistry

Ozonated water is widely used for local therapy in particular. Its positive effects are diverse: a disinfectant, anti-inflammatory activation of the intracellular metabolism of the oral mucosa and dental wounds; the advancement of local blood circulation; a provocation of regenerative functions; and a hemostatic effect in capillary bleeding [22]. The anti-inflammatory effect of ozone is based on its ability to oxidize compounds containing double bonds, including arachidonic acid and the prostaglandins synthesized from it, biologically active substances involved in developing and maintaining the inflammatory process in high concentrations [23,24]. Reactions with other derivatives of arachidonic acid, namely, leukotrienes, can partly explain the effect of ozone therapy in patients with bronchial asthma. Leukotrienes, as purely pathological compounds synthesized in leukocytes from arachidonic acid, provoke slow allergic reactions such as bronchial asthma attacks. In addition, ozone reduces the degree of tissue hypoxia and restores metabolic processes in the affected tissues at the site of inflammation, correcting pH and electrolyte balance.

Ozone has a pronounced analgesic effect, which can be associated with several points [1,22,23]: (1) the anti-inflammatory effect of ozone is due to its modulating effect on prostaglandins, which regulate cellular reactions (ozone prevents the modulation of the arachidonic acid cascade); (2) due to the increase in tissue oxygenation, the metabolism and elimination of products that cause the activation of pain receptors are enhanced; and (3) as a result of the increased release of oxygen in the tissues, the cation/anion ratio in the altered cell membrane is again enhanced, that is, ozone acts as an antagonist of pain.

In daily surgical dental practice, ozonated water is an extremely important therapeutic agent. When extracting a diseased tooth due to periodontitis or exacerbated apical processes, ozonated water is the means of choice, due to its disinfecting effect. Before the start of the intervention, for the purpose of disinfection, the oral cavity is rinsed with ozonated water, and then, the abscess cavity is intensively washed with the same solution after it is opened. Over the next 2-3 days, it is recommended to repeat this procedure until the wound cavity heals. Sen and Sen [25] state that ozonated water should be used at a temperature of 20 degrees with an output ozone concentration of 10-20 mg/L. Due to the local impact of ozone or its peroxides, immunocompetent cells in the mesenchyme are activated, and thus, allergic reactions in the pathologically altered bone are stopped.

Given the anti-inflammatory and immunomodulatory effects of ozone and its effect on microcirculation, ozone therapy is widely used in the conservative treatment of open mandibular fractures. After surgical intervention in the area of the fracture, the oral cavity is rinsed with ozonated distilled water with an ozone concentration of 1,500 µg/L daily for five days [26]. After rinsing in the oral fluid, an increase in antioxidant activity has been noted, which contributes to inhibiting lipid peroxidation processes and preventing inflammation. At the same time, patients receive ozonated saline solution (OSS) intravenously at an ozone concentration of 1,000 μg/L, which contributes to the disappearance of pain in the area of the mandibular fracture, and traumatic edema is eliminated on days 4-5 [27]. After a course of ozone therapy in patients with trauma to the lower jaw with no inflammatory fracture complications, consolidation occurs 3-6 days faster than with traditional treatment.

The surgical treatment of periodontopathy is conducted with the use of ozonized solutions. In patients with periodontitis, periodontal pockets are treated with trundles impregnated with OSS 2-3 days before surgery, and oral baths with the same solution are simultaneously used once a day [28]. In the postoperative period, OSS is used only in oral baths, and applications with ozonated oil are required to the suture line. The concentration of ozone to obtain OSS is 2-3 mg/L [29]. The local application of ozone favorably affects the postoperative course. Epithelialization is accelerated, which makes it possible to remove the sutures on the fifth day after the operation to reduce treatment time significantly.

In prosthetics, ozonated water is used as a rinse for several days after preparation to disinfect and stimulate metabolism in the sulcus area if damage to this crucial area has occurred. In conservative dentistry, ozonated water is used for disinfection during tooth cavity preparation. OSS with a concentration of 2 mg/L is used in the form of rinses at the stages of teeth cleaning to intensify and optimize professional oral hygiene [30]. These two complementary methods effectively affect the microbial landscape of the oral cavity.

## Conclusions

Ozone therapy is a promising alternative method for treating caries compared with the traditional method involving the injection of anesthesia and preparation of a carious cavity using a drill. When treating caries with this method, the doctor does not remove the infected tissue, as is done by drilling, but ozonizes the affected area, which helps keep the tooth intact. The triatomic oxygen molecule is a powerful and active oxidizing agent that quickly and effectively disinfects any surface in the oral cavity. The principle of operation of ozone is quite simple: it reacts with a bacterium or virus, penetrating through its shell, and destroys pathogenic microorganism cells from the inside. The gas is produced by a special apparatus in which a generator is built. The control, dosage, and accuracy of the ozone supply are provided automatically. Such dental treatment stops the development of caries for a long time. After treatment with ozone, the area is covered with a special substance, a reductant, which promotes rapid remineralization, normalization of the acid-base balance, and strengthening of the enamel.
